# The* In Vitro* and* In Vivo* Wound Healing Properties of the Chinese Herbal Medicine “Jinchuang Ointment”

**DOI:** 10.1155/2016/1654056

**Published:** 2016-04-20

**Authors:** Tsung-Jung Ho, Shinn-Jong Jiang, Guang-Huey Lin, Tzong Shiun Li, Lih-Ming Yiin, Jai-Sing Yang, Ming-Chuan Hsieh, Chun-Chang Wu, Jaung-Geng Lin, Hao-Ping Chen

**Affiliations:** ^1^Division of Chinese Medicine, China Medical University Beigang Hospital, Yulin 65152, Taiwan; ^2^Division of Chinese Medicine, An Nan Hospital, China Medical University, Tainan 70965, Taiwan; ^3^College of Chinese Medicine, China Medical University, Taichung 40421, Taiwan; ^4^Department of Biochemistry, School of Medicine, Tzu Chi University, Hualien 97004, Taiwan; ^5^Department of Microbiology, School of Medicine, Tzu Chi University, Hualien 97004, Taiwan; ^6^Department of Plastic Surgery, China Medical University Beigang Hospital, Yulin 65152, Taiwan; ^7^School of Medicine, China Medical University, Taichung 40421, Taiwan; ^8^Department of Plastic Surgery, China Medical University Hospital, Taichung 40447, Taiwan; ^9^Department of Public Health, School of Medicine, Tzu Chi University, Hualien 97004, Taiwan; ^10^Department of Medical Research, China Medical University Hospital, China Medical University, Taichung 40447, Taiwan; ^11^Graduate Institute of Chinese Medicine, China Medical University, Taichung 40421, Taiwan

## Abstract

“Jinchuang ointment” is a traditional Chinese herbal medicine complex for treatment of incised wounds. For more than ten years, it has been used at China Medical University Hospital (Taichung, Taiwan) for the treatment of diabetic foot infections and decubitus ulcers. Three different cases are presented in this study. “Jinchuang” ointment is a mixture of natural product complexes from nine different components, making it difficult to analyze its exact chemical compositions. To further characterize the herbal ingredients used in this study, the contents of reference standards present in a subset of the ointment ingredients (dragon's blood, catechu, frankincense, and myrrh) were determined by HPLC. Two* in vitro* cell based assay platforms, wound healing and tube formation, were used to examine the biological activity of this medicine. Our results show that this herbal medicine possesses strong activities including stimulation of angiogenesis, cell proliferation, and cell migration, which provide the scientific basis for its clinically observed curative effects on nonhealing diabetic wounds.

## 1. Introduction

 It is well-known that diabetic foot ulcers are extremely difficult to be treated and are the dominant complication leading to amputations [1]. “Jinchuang ointment” is a traditional Chinese herbal medicine complex for treatment of incised wounds. Its recipe was first described in one ancient Chinese book of medicine,* Medicine Comprehended*, published in 1732. Clinical applications of this herbal medicine for diabetic foot infections and decubitus ulcers have been a successful course of treatment in the Division of Chinese Medicine, China Medical University Hospital, Taichung, Taiwan, for more than ten years. Despite its track record of curative effects, there is no literature published in the English language describing the clinical efficacy of “Jinchuang ointment” [2]. Moreover, neither a biological mechanism nor the compositions of effective components have yet to be systematically investigated. Like many Chinese herbal medicines, “Jinchuang ointment” is also a mixture of natural product complexes. The combination of compounds results in complications when it determines the chemical composition and bioactivity of each component [3].

“Jinchuang ointment” is composed of lard, wax, starch, synthetic borneol, camphor, frankincense, dragon's blood, myrrh, and catechu. To further characterize the chemical content of each component in this complex, the ratio of stereoisomers in chemically synthesized borneol used in this study was analyzed by chiral gas chromatography (GC). Meanwhile, the content of reference standards in the herbal components, like frankincense, myrrh, dragon's blood, and catechin, was determined by high performance liquid chromatography (HPLC). Lard is the major component in this complex, and its weight percentage is as high as 67%. It is of great interest to determine the role of lard in this complex. Lard was therefore substituted for synthetic triacylglycerol, coconut oil, Vaseline®, and sesame oil. The activity of these reconstituted complexes was examined in this study.

“Jinchuang ointment” is directly applied to the wound surface during treatment. The components in this complex are neither digested nor absorbed in the gastrointestinal tract. It is therefore reasonable to evaluate its bioactivity by direct addition of this complex into media containing cultured human skin or endothelial cells. Wound healing is a very complicated process. In this study, an* in vitro* tube formation assay, a wound healing assay, and a cell proliferation test were carried out to examine the activity of “Jinchuang ointment.” Here, we report the outcomes of treating patients with “Jinchuang ointment,” the results of cell based activity assays, and characterization of herbal components by HPLC.

## 2. Materials and Methods

### 2.1. Materials

The reference standards of dracorhodin perchlorate, acetyl-11-keto-*β*-boswellic acid, catechin, and epicatechin were purchased from the National Institute for the Control of Pharmaceutical and Biological Products (Beijing, China). (E)-Guggulsterone and (+)-borneol were obtained from Sigma-Aldrich (St. Louis, MO, USA). (−)-Borneol and (±)-isoborneol were purchased from Alfa Assar (Lancashire, UK). DMEM high glucose with sodium pyruvate media, Catalog number: I-26F55-I, was bought from Amimed® BioConcept (Allschwil, Switzerland). Chemically synthesized borneol and camphor were bought from Cheng Yi Chemical Co., Ltd. (Taipei, Taiwan). Herbal medicine, dragon's blood, catechu, frankincense, and myrrh were bought from Healthy Beautiful Biotech. Co. Ltd. (Taichung, Taiwan). Vascular endothelial growth factor (VEGF) was bought from B&D Systems (Minneapolis, MN, USA). Antibodies for western blot analysis were bought from Santa Cruz Biotechnology, Inc. (Dallas, Texas, USA). Food-grade lard was obtained from I-Mei Food Company (Taipei, Taiwan). Synthetic triacylglycerol (glyceryl tricaprylate-caprate, C_8_ : C_10_, 60 : 40) was obtained from InterMed Manufacturing Sdn. Bhd. (Kuala Lumpur, Malaysia). Food-grade sesame oil was from Fwusow Industry Co. Ltd. (Taichung, Taiwan). Coconut oil was bought from First Chemical Co., Ltd. (Taipei, Taiwan). Vaseline was bought from Unilever (Trumbull, Connecticut, USA).

The composition of “Jinchuang ointment” (100 g) is as follows: lard 67.3 g, dragon's blood 2.1 g, catechu 2.1 g, frankincense 2.1 g, myrrh 2.1 g, camphor 6.3 g, borneol 0.1 g, corn starch 8.4 g, and wax 9.5 g. For wound healing and tube formation assays, the DMSO stock solution of “Jinchuang ointment” is prepared as follows: two grams of “Jinchuang ointment” is dissolved in 10 mL DMSO and homogenized by ultrasonication just before use.

### 2.2. Determination of Reference Standard Content in Dragon's Blood, Catechu, Frankincense, and Myrrh by HPLC

All experiments were carried out on a Hitachi L-7000 HPLC system, equipped with L-7100 quaternary gradient pump and a L-7450 photo diode array detector. Hitachi HSM software was used for machine controlling, data collecting, and processing. A Mightysil, RP-18, 5 *μ*m, 250 × 4.6 mm, analytic column (Kanto Chemical Co., Inc., Tokyo, Japan) was used for analysis.

For samples of dragon's blood, catechu, and frankincense, 0.1 g grounded solids were weighed and dissolved in 10 mL of methanol. After ultrasonicating for 30 minutes at room temperature, methanol extracts were transferred to a new glass vial by using disposable glass Pasteur pipettes. 4 mL of methanol was then added and ultrasonicated for another 30 minutes at room temperature. The final volume of the extract was adjusted to 25 mL by adding methanol. Undissolved particles were removed by centrifugation at 2500 ×g for 10 minutes at room temperature and filtrated through a 0.22 *μ*m syringe filter. For myrrh, 95% ethanol was used for extraction rather than methanol. Other preparation steps were identical to those of dragon's blood, catechu, frankincense, and myrrh.

The methanol extract of dragon's blood was separated using a gradient elution of solvent A (10% CH_3_CN containing 0.1% formic acid) and solvent B (90% CH_3_CN containing 0.1% formic acid) with a flow rate of 1 mL/min [4]. The elution program is given in Table 1. The UV detection wavelength was 254 nm.

The catechu methanol extract was separated using a gradient elution of A (H_2_O containing 0.1% formic acid), B (10% CH_3_CN containing 0.1% formic acid), and C (90% CH_3_CN containing 0.1% formic acid) with a flow rate of 1 mL/min [5]. The elution program is given in Table 1. The UV detection wavelength was 270 nm.

The frankincense methanol extract was separated using a gradient elution of solvent A (9.5% methanol containing 0.5% H_3_PO_4_, 85%) and solvent B (45% methanol, 55% CH_3_CN containing 0.5% H_3_PO_4_, 85%) with a flow rate of 1 mL/min [6]. The elution program is given in Table 1. The UV detection wavelength was 250 nm.

To determine the content of (E)-guggulsterones in myrrh, an ethanol extract was separated using an isocratic elution of 0.1% H_3_PO_4_ : CH_3_CN (45 : 55, v/v) with a flow rate of 1 mL/min for 25 minutes [7]. The UV detection wavelength was 240 nm.

### 2.3. Determination of the Ratio of Stereoisomers in Synthetic Borneol

Chiral GC was used to determine the ratio of stereoisomers in synthetic borneol. Analysis was performed using an Agilent GC system model HP 6890N (Santa Clara, CA, USA) equipped with a split/splitless injector, liner of silanized quartz with a 4 mm i.d. (effective volume 0.49 mL), and an Agilent 6890 autosampler for 100 vials. A Cydex-B chiral GC column (25 m × 0.22 mm ID; 0.25 *μ*m) was obtained from SGE Analytical Science (Austin, TX, USA). Chromatographic conditions were as follows: helium used as a gas carrier; a constant flow of 1.0 mL/min; 2 *μ*L injection volume (splitless model), and a 280°C injector temperature. The GC temperature program was as follows: 90°C (1 min), 90°C to 130°C (41 min), 130°C (10 min), and 200°C (3 min). An Agilent 5973N quadrupole mass spectrometer was operated in selective ion monitoring (SIM) mode, with ionization source by electron impact at 70 eV, transfer line at 280°C, ion source at 280°C, and quadrupole at 150°C.

### 2.4. *In Vitro* Wound Healing Assay

Confluent HaCaT cells in 12-well plates were starved overnight in DMEM medium. The surface of the plate was scraped with a 200 *μ*L pipette tip to generate a cell-free zone. Free cells were then removed by two HBSS washes, and cells were incubated in DMEM medium containing 200 *μ*g/mL, 20 *μ*g/mL, or 2 *μ*g/mL “Jinchuang ointment.” After 24 hours of incubation, cells were imaged using microscopy. The area of wound closure was quantitatively determined using Image J software (National Institutes of Health, Bethesda, MD).

Stimulation effects on* in vitro* wound healing assay by “Jinchuang ointment” were also examined using human microvascular endothelial cells (HMEC-1). Ibidi Culture-Inserts (Ibidi Gmbh, Martinsried, Germany) were placed on the chamber of 24-well cell culture plates. About 70 *μ*L of HMEC-1 (5 × 10^5^ cells/mL) was seeded per well and plates were incubated at 37°C and 5% CO_2_. After 24-hour incubation, Ibidi Culture-Inserts were removed, and 1 mL of MCDB131 media containing 1 *μ*L DMSO solution of “Jinchuang ointment” was then added into individual wells. The migration of cells was observed by microscopy over a period of 6–24 hours. The gap size was measured by using software Image J.

### 2.5. Cell Proliferation Assay

HaCaT cells (5 × 10^4^/well) were seeded in 96-well plates. The medium containing “Jinchuang ointment” at various concentrations was then added after cell adhesion. Cells were incubated in DMEM medium for the indicated time. The proliferation of HaCaT cells was subsequently determined by using Cell Proliferation Reagent WST-1 (Roche, Indianapolis, IN, USA). The statistical method used is Student's *t*-test.

### 2.6. Western Blotting

Confluent monolayers of HaCaT cells were treated with various concentrations of Jinchuang ointment for the indicated time. Equal quantities of cell lysate proteins were separated by 10% SDS-PAGE and electroblotted onto PVDF membranes (Millipore, Billerica, MA). Membranes were blocked for 1 h with 5% low-fat milk powder solubilized in phosphate-buffered saline (PBS) containing 0.05% Tween 20. Levels of Cdc25b, Cdc25c, CDK2, CDC D2, Cyclin B, Cyclin D3, and *α*-tubulin were determined by western blotting using specific antibodies and enhanced chemiluminescence detection methods. The intensity of the resulting bands was measured by densitometric analysis using Image J software and presented as the ratio relative to the internal control.

### 2.7. *In Vitro* Tube Formation Assay

Human umbilical vein endothelial cells (HUVEC) were bought from Bioresource Collection and Research Center (Hsinchu, Taiwan). 1 mL of HUVEC (1 × 10^5^ cells/well) was placed into the wells of a 24-well flat bottomed plates precoated with 200 *μ*L Matrigel (BD Biosciences, Bedford, MA, USA). Cells were then mixed with 1 mL medium containing “Jinchuang ointment” (final concentration of 200 *μ*g/mL). Cells were incubated at 37°C for a 24 h exposure [8]. After incubation, cell tube or network formation was observed using a phase-contrast microscope.

### 2.8. Statistical Analysis

Results are expressed as the means ± SD from at least three independent experiments. Differences between groups were assessed by one-way analysis of variance (ANOVA). A *P* value less than 0.05 was considered statistically significant.

## 3. Results and Discussion

### 3.1. Clinical Treatment Observations on a Nonhealing Diabetic Wound by Treating with “Jinchuang Ointment”

**Figure 1:**
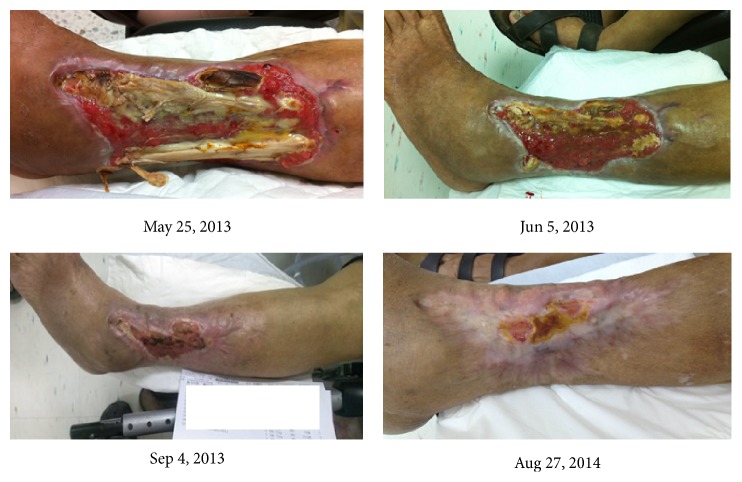
The wound area of the patient Mrs. Wu during “Jinchuang ointment” treatment. Wound dimensions measured on the date specified are as follows: May 25, 2013, 26 × 9 cm; Jun 5, 2013, 26 × 9 cm; Sep 4, 2013, 8 × 4 cm; Aug 27, 2014, 2.5 × 3 cm.

To the best of our knowledge, there are no English language case reports describing “Jinchuang ointment” treatment for nonhealing diabetic wounds. Three different cases are presented in this study. The first subject, Mrs. Wu, is a 75-year-old female patient with type II insulin-dependent diabetes accompanied by peripheral arterial occlusion disease (PAOD) which led to left lateral leg and ankle necrotizing fasciitis. She was treated with percutaneous transluminal angiography (PTA) to improve lower limb circulation on Feb 6, 2013. As a result of reperfusion injury, the ulcer had enlarged with erythema. After examination, a below-knee amputation was immediately scheduled two weeks later at the Surgery Division, the China Medical University Beigang Hospital. As suggested by a doctor from the Division of Chinese Medicine, she decided to use traditional Chinese medicine to treat her wound. She was referred to the Division of Chinese Medicine for wound management. Normal saline was first used to clean the wound. About 2-3 g “Jinchuang ointment” was applied directly to the wound once daily. Pictures depicting wound healing under treatment with “Jinchuang ointment” are shown in Figure 1.

**Figure 2:**
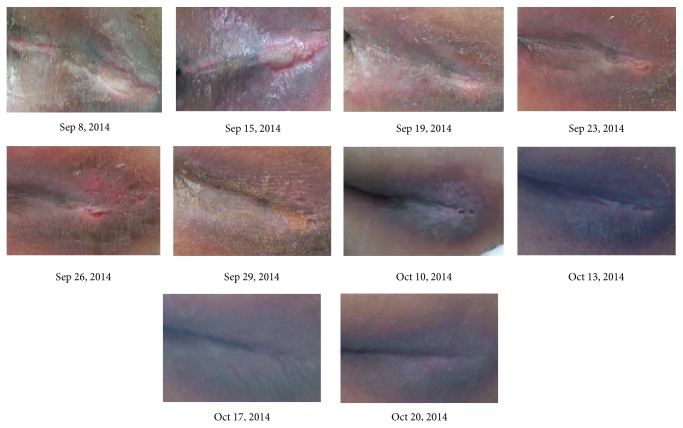
The pressure sore of the patient Mr. Tsai during “Jinchuang ointment” treatment. This wound was located at the sacral region and was completely closed after a 50-day treatment.

Mr. Tsai, a 71-year-old male, with past history of type II diabetes was diagnosed on September 8, 2013. He had a 3 × 0.8 cm and a 4 × 1.5 cm grade 3 pressure sore in the sacral region. 1 g of “Jinchuang ointment” was applied topically to the wound area once per day. Pictures documenting wound healing under treatment of “Jinchuang ointment” are shown in Figure 2. It is well-known that all treatments for bedsores are to prevent wounds from worsening. Complete wound closure was observed on October 27, 2013.

**Figure 3:**
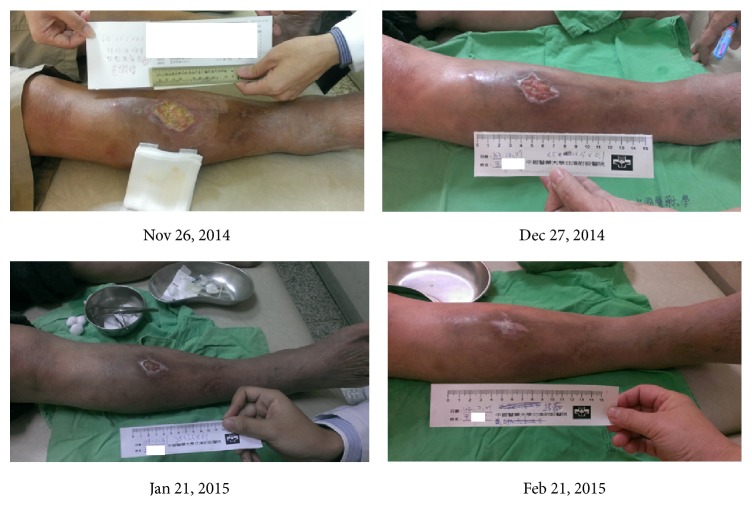
The previously nonhealing wound of patient Mr. Wang during “Jinchuang ointment” treatment. Wound dimensions were measured on the following dates: Nov 26, 2014, 6.2 × 5.3 × 0.3 cm; Dec 27, 2014, 4.5 × 4 × 0.1 cm; Jan 21, 2015, 2.5 × 1.5 × 0.1 cm; Feb 21, 2015, wound complete closure.

Mr. Wang, a 64-year-old male with a past history of hypertension, had a chronic wound measuring 6.2 × 5.3 cm which had not healed for more than six months. He received the topical application of “Jinchuang ointment” once per day beginning November 26, 2014. A great improvement was observed after two months of treatment as shown in Figure 3.

### 3.2. Content of Reference Standards Present in Dragon's Blood, Catechu, Frankincense, and Myrrh

One of the main problems associated with herbal medicine is the high level of batch to batch variation in the amounts of active components. The content of pure chemical reference standards in herbal products is therefore used as an indicator for the purposes of quality control and standardization. Accordingly, the content of reference standards in dragon's blood, catechu, frankincense, and myrrh used was measured by HPLC in this study. All the calibration curves of reference compounds were linear over the concentration range studied (Table 2). A linear interpolation method was used to calculate the percentage by mass of each reference standard in the herbal extract that we examined.

**Figure 4:**
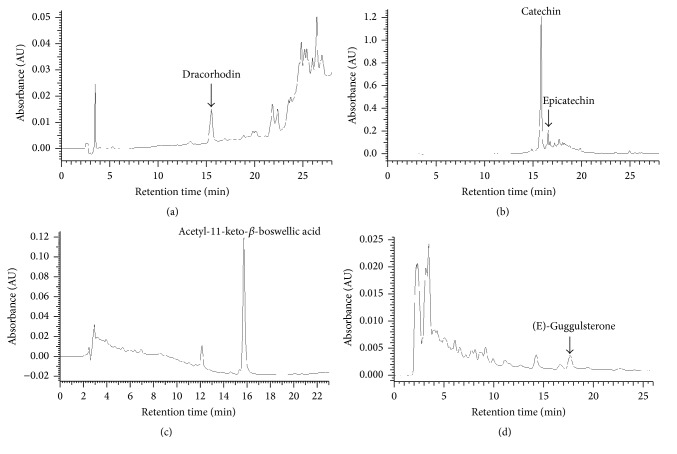
HPLC separation of reference compounds present in extracts of herbal components. HPLC traces of (a) dracorhodin in “dragon's blood,” (b) catechin and epicatechin in catechu, (c) acetyl-11-keto-*β*-boswellic acid in frankincense, and (d) (E)-guggulsterone in myrrh.

Dracorhodin is a red anthocyanin pigment that is a major component in “dragon's blood” resin of the plant* Daemonorops draco*. It possesses antimicrobial, anticancer, and cytotoxic activity [9, 10].  Figure 4(a) shows the separation of dracorhodin in dragon's blood. The mass percentage of dracorhodin in the “dragon's blood” used in this study is 0.15%.

Both catechin and epicatechin are phenol-type antioxidants in catechu, an extract of acacia trees. Catechu is a common component of herbal medicine. In addition to their ability to scavenge free radicals in plasma, the health benefits of catechin and epicatechin also include stimulation of fat oxidation, expansion of the brachial artery, and resistance of LDLs to oxidation [11]. At the cellular and molecular level, catechin can enhance the expression of human PTGS2 (a dioxygenase gene), 1L1B (cyclooxygenase-2 gene), SOD (superoxide dismutase gene), MAPK1 (Mitogen-activated protein kinase 1), and MAPK3 [12–14].  Figure 4(b) shows the separation of catechin and epicatechin in catechu. The mass percentage of catechin and epicatechin in the catechu used in this study is 24.2% and 1.7%, respectively.

Frankincense is a resin from plants in the genus* Boswellia*. In Africa and Asia, it is widely used in incense, perfume, and traditional medicine. Boswellic acids, a series of pentacyclic triterpene molecules, are one of the major components of frankincense. Theiranti-inflammatory properties and ability to induce cancer cell apoptosis have been reported* in vitro* [15–17]. The expression of TOP1 (DNA topoisomerase I) and TOP2A (DNA topoisomerase II) genes can be altered in the presence of 11-keto-*β*-boswellic acid derivatives and acetyl-boswellic acid [16, 18].  Figure 4(c) shows the separation of acetyl-11-keto-*β*-boswellic acid in frankincense. The mass percentage of acetyl-11-keto-*β*-boswellic acid in the frankincense used in this study is 1.62%.

Myrrh is also a resin from plants in the genus* Commiphora*. The usage of myrrh is similar to that of frankincense. In fact, both myrrh and frankincense are frequently used in concert in many traditional Chinese medicine recipes. In western medicine, myrrh is also used in liniment and healing salves for minor skin ailments. The chemical composition of myrrh is rather complicated [19]. Notably, both (Z)- and (E)-isomers of guggulsterone possess high affinity toward a variety of steroid receptors [20]. Both isomers seem equipotent as inhibitors of HUVEC tube formation [21]. The mass percentage of (E)-guggulsterone in the myrrh used in this study is 0.02%.  Figure 4(d) shows the separation of (E)-guggulsterone in myrrh.

### 3.3. The Ratio of Stereoisomers in Chemically Synthesized Borneol

**Figure 5:**
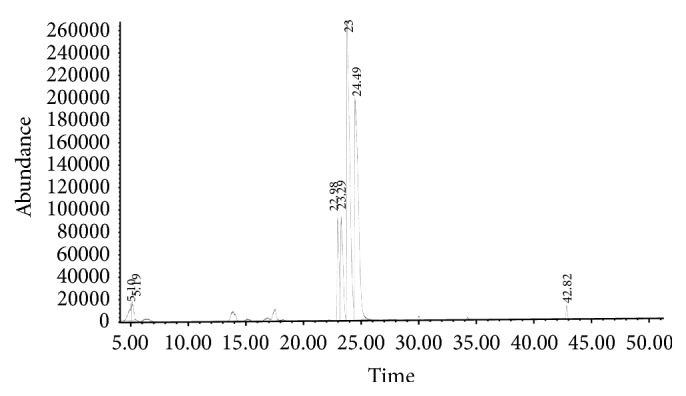
Chiral GC separations of synthetic borneol. The four major peaks from 22.0 to 26.0 min are (+)-isoborneol, (−)-isoborneol, (−)-borneol, and (+)-borneol, respectively.

Borneol is a bicyclic monoterpene plant secondary metabolite. (+)-Borneol, mainly isolated from the plant family of Dipterocarpaceae, is the major form used for analgesia and anesthesia in traditional Chinese medicine. More recently, chemically synthesized borneol and (−)-borneol isolated from the plant species* Blumea balsamifera* have enjoyed more widespread use. The price of synthetic borneol is the lowest among the sources discussed above. Previous results show that borneol stereoisomers can interact with GABA receptors [22, 23] and possess antimicrobial activity [24]. Chiral GC was used to analyze the composition of borneol stereoisomers in synthetic borneol used in this study. Our results show that (+)-isoborneol : (−)-isoborneol : (−)-borneol : (+)-borneol is in the ratio of 8.9 : 10.9 : 41.6 : 38.6 (Figure 5).

### 3.4. *In Vitro* Wound Healing Assay

**Figure 6:**
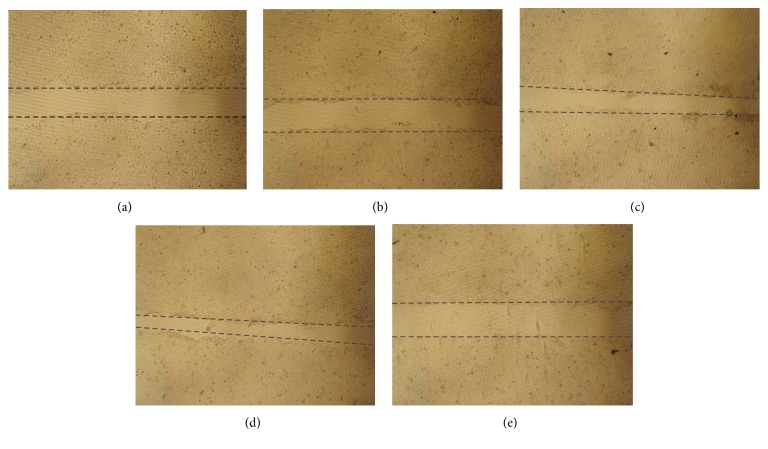
Wound healing assay with HaCaT cells displaying the increased cell migration induced by “Jinchuang ointment.” Cells were treated with (a) DMSO alone (control), (b) 100 ng/mL EGF (positive control), (c) 200 *μ*g/mL “Jinchuang ointment,” (d) 20 *μ*g/mL “Jinchuang ointment,” and (e) 2 *μ*g/mL “Jinchuang ointment.” Cell migration was documented by phase contrast microscopy over a 24-hour time course where time 0 is the time of wound scratching.

Confluent HaCaT cells were scratched and treated with “Jinchuang ointment.” The wound area was measured after 24 hours of treatment. All experiments were performed in triplicate. The percentage of wound closure was calculated as follows: (initial wound area − 24 h posttreatment wound area)/initial wound area × 100%. The wound closure percentage with 200 *μ*g/mL, 20 *μ*g/mL, and 2 *μ*g/mL “Jinchuang ointment” treatment was 78.2 ± 5.3%, 78.2 ± 4.3%, and 63.8 ± 1.7%, respectively. In contrast, the negative control HaCaT cells without “Jinchuang ointment” treatment only showed 64.4 ± 4.0% wound closure. The positive control treated with 100 ng/mL EGF treatment showed 71.8 ± 1.0% wound closure. It is obvious that application of 200 *μ*g/mL or 20 *μ*g/mL “Jinchuang ointment” is more potent than 100 ng/mL EGF in promoting* in vitro* wound closure, showing statistically significant differences when compared to both positive and negative controls (Figure 6).

**Figure 7:**
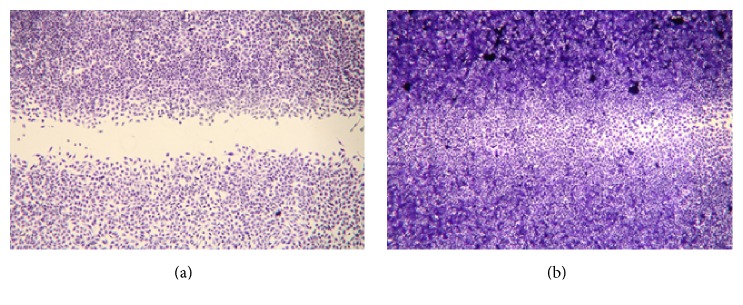
Wound healing assay with HMEC-1 cells displaying the increased cell migration induced by “Jinchuang ointment.” Cells were treated with (a) DMSO alone (control) or (b) 200 *μ*g/mL “Jinchuang ointment.” Cell migration was recorded and cells were stained by microscopy over a 24-hour time course.

**Figure 8:**
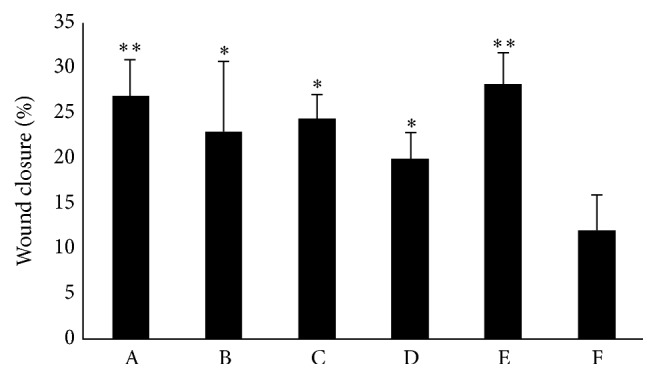
The percentage of wound closure with HMEC-1 cells after six hours of treatment in response to reconstituted variants “Jinchuang ointments.” Lard is replaced by sesame oil (group B), synthetic triacylglycerol (group C), coconut oil (group D), and Vaseline (group E). Group A is the original recipe of Jinchuang ointment, and group F is DMSO only (control). Values are the mean ± SD. ^*∗*^ *P* < 0.05 and ^*∗∗*^ *P* < 0.01 compared with control.

The wound-healing assay was also used to assess the stimulatory effect of “Jinchuang ointment” on the migration of HMEC-1 cells. The wound closure percentage with 200 *μ*g/mL “Jinchuang ointment” 24 hours after treatment was 85.0 ± 12.3%, whereas the wound closure percentage observed with the DMSO control was only 43.3 ± 8.2% (Figures 7(a) and 7(b)). The weight percentage of lard is as high as 67% in “Jinchuang ointment,” and sesame oil is used to prepare another famous traditional Chinese herbal ointment, Shiunko. The contribution of lard to the total cell migratory activity was therefore evaluated by reconstituting “Jinchuang ointment” with various fats. It is apparent that the significant cell migration into the wound region is seen with Jinchuang ointment-treated cells when compared to the control group (Figure 8). When sesame oil, synthetic triacylglycerol, coconut oil, and Vaseline were used as lard substituents, 85%, 91%, 74%, and 105% migration activity can be observed (Figure 8). Unlike natural fat, Vaseline is mainly made from petroleum jelly, a semisolid mixture of hydrocarbons with carbon numbers greater than 25. The chain length of carbon atoms in the synthetic triacylglycerols used in this study is C_8_ and C_10_. These results suggest that the carbon chain length of the fat used in the ointment plays a minor role in stimulating cell migration.

### 3.5. The Effect of “Jinchuang Ointment” on HaCaT Cell Proliferation

**Figure 9:**
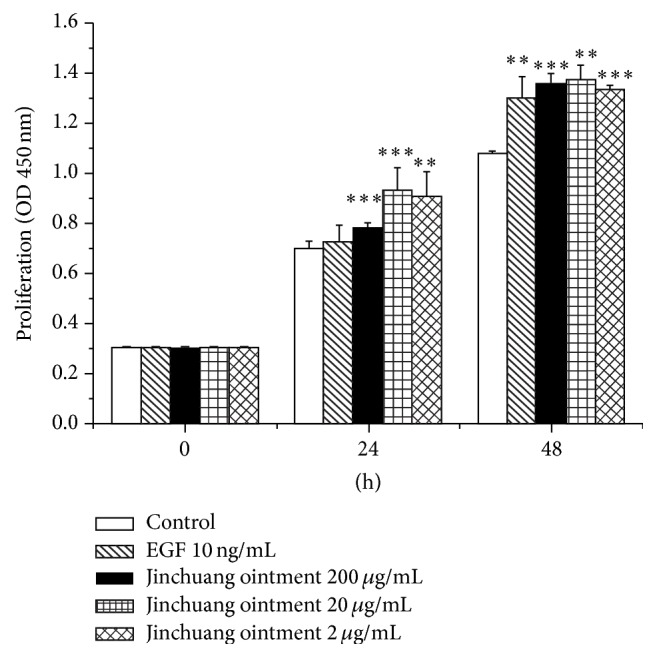
Proliferation of HaCaT cells measured by a WST-1 assay. Increased cell proliferation of HaCaT cells was observed in the presence of 200 *μ*g/mL, 20 *μ*g/mL, and 2 *μ*g/mL “Jinchuang ointment.” Negative and positive controls are in the presence of DMSO and 10 ng/mL EGF, respectively. Values are the mean ± SD. ^*∗∗*^ *P* < 0.01 and ^*∗∗∗*^ *P* < 0.001 compared with control.

The stimulatory effect of “Jinchuang ointment” on HaCaT cell proliferation was evaluated in the presence of 10% fetal bovine serum (FBS) by WST-1 assay. The increased percentages of cell proliferation by 200 *μ*g/mL, 20 *μ*g/mL, and 2 *μ*g/mL “Jinchuang ointment” at 24 and 48 hours were 117 ± 2.66%, 133 ± 12.7%, and 129.7 ± 14.1% and 126.4 ± 3.5%, 127.3 ± 5.8%, and 124.1 ± 1.3%, respectively (Figure 9). These results indicate that treatment with “Jinchuang ointment” leads to an increase in HaCaT cell growth.

### 3.6. The Effect of “Jinchuang Ointment” on the Expression of G1/S Transition-Related Regulators

**Figure 10:**
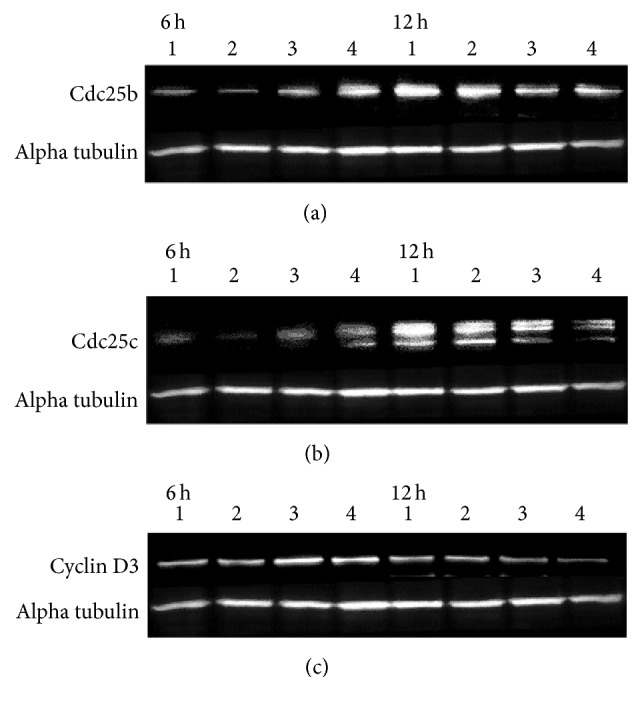
Treatment of HaCaT cells with “Jinchuang ointment” leads to altered expression of cell cycle-related proteins.Western blot analysis of (a) Cdc25b, (b) Cdc25c, and (c) cyclic D3 protein expression in HaCaT cell extracts after six and 12 hours of treatment. Lanes one to four are as follows: control, 100 ng/mL EGF, 200 *μ*g/mL “Jinchuang ointment,” and 20 *μ*g/mL “Jinchuang ointment,” respectively.

To further investigate the underlying mechanisms involved in “Jinchuang ointment”-induced effects on cell proliferation in HaCaT cells, the expression of several key cellular proteins involved in cell cycle progression was investigated by western blot analysis. As shown in Figure 10, a six-hour treatment with “Jinchuang ointment” leads to a dose-dependent increase in Cdc25b, Cdc25c, and Cyclin D3 levels. After treatment for 12 hours, significant changes in the expression pattern of those proteins between experiment and control groups were observed, suggesting that “Jinchuang ointment” alters HaCaT cell cycle progression.

### 3.7. Tube Formation Assay

**Figure 11:**
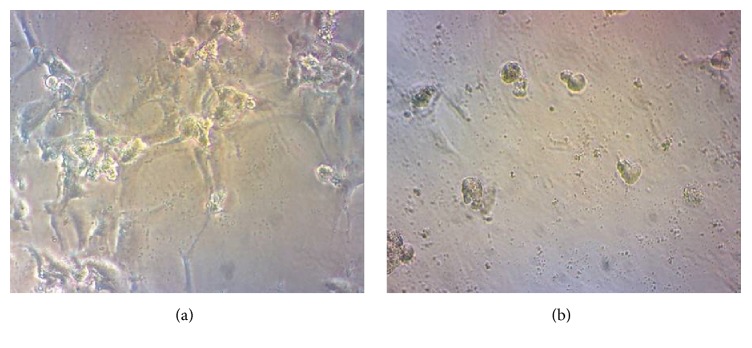
*In vitro* tube formation assay displaying the stimulation of angiogenesis by “Jinchuang ointment” in HUVEC cells. Cells were treated with (a) 200 *μ*g/mL “Jinchuang ointment” and (b) DMSO only (negative control).

The process of wound healing has been divided into three different stages, inflammatory, proliferative, and remodeling phases [25]. Angiogenesis is responsible for new blood vessel formation and oxygen and nutrient supply and plays an important role in the proliferative phase of wound healing [26]. The tube formation assay was used to evaluate the* in vitro* angiogenic effect of “Jinchuang ointment” on HUVEC cells. As shown in Figure 11, treatment of HUVEC cell with 200 *μ*g/mL “Jinchuang ointment” for 24 hours can efficiently induce endothelial cell capillary tubes and network formation.

### 3.8. Conclusions

“Jinchuang ointment” is a Chinese herbal medicine complex. It has been clinically used in the treatment of diabetic foot infection and decubitus ulcers in China Medical University Hospital for more than ten years. Because of its complicated composition, its biological activities have never been investigated. To further characterize its herb ingredients, the content of reference standards present in dragon's blood, catechu, frankincense, and myrrh was determined by HPLC. Two cell based assay platforms,* in vitro* wound healing and tube formation, were used to examine activity. Our results show that this herbal medicine possesses potent activities stimulating cell proliferation, migration, and angiogenesis. This provides a scientific rationale to account for the observed clinical curative effects on wound healing by “Jinchuang ointment.”

According to current pharmaceutical regulations in Taiwan, only traditional Chinese herbal medicine manufactured from cGMP pharmaceutical factories can be sold in drug stores or by hospital marketing channels. However, for homemade traditional Chinese herbal medicine, they can only be administered to patients by the doctors who made the respective medicine. For this reason, “Jinchuang ointment” cannot be widely used in the Taiwan area since many clinicians are not able or unwilling to prepare this remedy. To facilitate the manufacturing process of “Jinchuang ointment” by cGMP factories, it will be very important to find out the activity indicator markers for components, such as dragon's blood, catechu, frankincense, and myrrh.

## Figures and Tables

**Table 1:** HPLC elution program for dragon's blood, catechu, and frankincense.

Dragon's blood		Catechu		Frankincense	
Time (min)	Eluent (B%)	Time (min)	Eluent (B, C%)	Time (min)	Eluent (B%)
0–2	19	0–5	0,0	0–5	90
2–20	19–100	5–7	0,0–100,0	5–11	90–100
20-21	100	7–12	100,0–20,80	11–23	100
		12–26	20,80–0,100		

**Table 2:** HPLC calibration curves of reference compounds including regression equations, the coefficients of determination (*R* ^2^), and calibration ranges.

Reference compound	Regression equation	*R* ^2^	Calibration range	Mass percentage
Dracorhodin perchlorate	*y* = 2000000*x* − 19368	0.999	0.0125–0.2 *μ*g	0.15%
Catechin	*y* = 26508*x* − 35003	0.997	0.25–4 *μ*g	24.2%
Epicatechin	*y* = 33500*x* − 65614	0.999	0.5–4 *μ*g	1.7%
Acetyl-11-keto-*β*-boswellic acid	*y* = 79427*x* − 54832	0.999	0.125–2 *μ*g	1.62%
(E)-Guggulsterone	*y* = 2466634*x* + 12554	0.999	0.0625–1 *μ*g	0.02%
